# Role of Peroxisome Proliferator-Activated Receptors (PPARs) in Energy Homeostasis of Dairy Animals: Exploiting Their Modulation through Nutrigenomic Interventions

**DOI:** 10.3390/ijms222212463

**Published:** 2021-11-18

**Authors:** Faiz-ul Hassan, Asif Nadeem, Zhipeng Li, Maryam Javed, Qingyou Liu, Jahanzaib Azhar, Muhammad Saif-ur Rehman, Kuiqing Cui, Saif ur Rehman

**Affiliations:** 1 State Key Laboratory for Conservation and Utilization of Subtropical Agro-Bioresources, Guangxi University, Nanning 530005, China; f.hassan@uaf.edu.pk (F.-u.H.); zp.li@gxu.edu.cn (Z.L.); qyliu-gene@gxu.edu.cn (Q.L.); 2 Institute of Animal and Dairy Sciences, Faculty of Animal Husbandry, University of Agriculture, Faisalabad 38040, Pakistan; shsaifurrehman@yahoo.com; 3 Department of Biotechnology, Virtual University of Pakistan, Lahore 54000, Pakistan; asif.nadeem@vu.edu.pk (A.N.); jahanzaib.azhar@vu.edu.pk (J.A.); 4 Institute of Biochemistry and Biotechnology, University of Veterinary and Animal Sciences Lahore, Lahore 54000, Pakistan; Maryam.javed@uvas.edu.pk

**Keywords:** nuclear receptors, PPARs, nutrigenomics, energy homeostasis, dairy animals

## Abstract

Peroxisome proliferator-activated receptors (PPARs) are the nuclear receptors that could mediate the nutrient-dependent transcriptional activation and regulate metabolic networks through energy homeostasis. However, these receptors cannot work properly under metabolic stress. PPARs and their subtypes can be modulated by nutrigenomic interventions, particularly under stress conditions to restore cellular homeostasis. Many nutrients such as polyunsaturated fatty acids, vitamins, dietary amino acids and phytochemicals have shown their ability for potential activation or inhibition of PPARs. Thus, through different mechanisms, all these nutrients can modulate PPARs and are ultimately helpful to prevent various metabolic disorders, particularly in transition dairy cows. This review aims to provide insights into the crucial role of PPARs in energy metabolism and their potential modulation through nutrigenomic interventions to improve energy homeostasis in dairy animals.

## 1. Introduction

Dairy animals provide milk and dairy products, which are considered some of the most important sources of nutrients for the human diet globally. Dairy production is the key element of sustainable agriculture in the tropics and subtropics. The rapidly increasing human population urges for consolidated efforts to ensure the abundant future availability of milk and dairy products. Therefore, problems and challenges associated with milk production and dairy animal health should be addressed to enhance the production of animals. Dairy animals experience diverse types of stress at different production stages in which the transition period is one of the most stressful stages in the life of dairy cattle. During the transition period and other stressful stages, the metabolic health of the animal is compromised, resulting in enhanced production of non-esterified fatty acids (NEFA) and ketone bodies (kb). Other major conditions associated with these stress conditions include insulin resistance, low blood sugar levels and inflammation [1], which lead to toxicity, fatty liver, ketosis and other metabolic syndromes, ultimately reducing the performance of dairy animals [2].

Nuclear receptors are known to regulate physiological events of metabolism and control the homeostasis of glucose and lipid metabolism. They are also implicated in mediating the long-term effects of early environmental and nutritional experiences on the onset of chronic metabolic disorders in humans and animals [3]. Nuclear receptors belong to a family of ligand-regulated transcription factors that are activated by steroid hormones, such as progesterone, estrogen, and different other lipid-soluble signals such as oxysterols, thyroid hormone, and retinoic acid [4]. In contrast to other messengers, ligands are one of the intercellular messengers that can cross the plasma membrane barrier and directly interact with nuclear receptors instead of interacting with cells surface receptors. These nuclear receptors, once activated, can directly regulate the transcription of respective genes and control many biological processes, including the reproduction, development, proliferation of cells, and metabolism. Despite the fact that the nuclear receptors primarily work as transcription factors, but some have additionally been found to regulate the function of cells inside the cytoplasm [5]. More than 50 nuclear receptors are being reported in human genomes [4,6]. Ligands for these have been discovered, except for a few “orphan receptors” [7]. Major nuclear receptors with more comprehensive experimental data and their ligands are summarized in Table 1.

**Figure 1:**
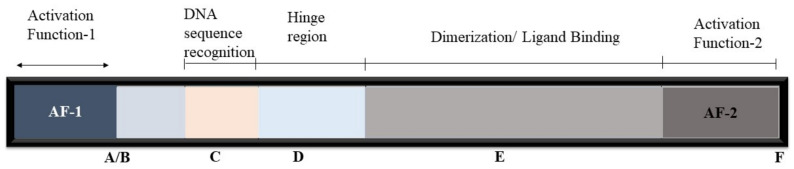
General basic association of atomic receptors [9]. Letters from A to F represented the domains of nuclear receptors from N-end to C-end.

All of the nuclear receptors have a common structure comprised of the highly variable amino-terminal domain that incorporates a few particular regions of transactivation (the A/B domain, also referred to as AF1 for activation function 1), a central conserved DNA-binding domain that contains two Zn fingers (the C domain), a short region responsible for nuclear localization (the D domain), and a large fairly well-conserved carboxy-terminal ligand-binding domain (the E domain, or LBD) that contributes to interactions of the subset of nuclear receptors that form heterodimers [4]. Further, a highly variable carboxy-terminal tail (the F domain) that in most cases has unknown functions is also present, as shown in Figure 1.

Research studies on metabolic syndromes have identified a close connection between metabolic abnormalities and nuclear receptors, including PPARs, farnesoid X receptors (FXRs), liver X receptors (LXRs) and glucocorticoid receptors (GRs) [3]. PPARs are widely studied nuclear receptors that are known to regulate and control metabolic changes both in humans and animals. Essentially, PPARs were first identified as novel members of the nuclear receptors from *Xenopus* frogs [10] and exhibited to induce the multiplication of peroxisomes in the cells. The PPARα was the first member of these receptors that was identified in mammals during the analysis of molecular targets for liver peroxisome proliferators [11]. The characterization of the PPARA (encoding PPARα) gene in adult mice revealed that PPARα found in humans and dairy animals is abundantly expressed in the liver, heart and kidney. After the discovery of PPARα, the other isotypes were also discovered, including PPARγ and PPARβ/δ [10]. The PPARs form heterodimers and function with the retinoid-X-receptor (RXR). Once a specific ligand binds to receptor dimer, it induces the covalent modification in the structure of PPARs, which activates these nuclear receptors [12]. The activated dimer PPAR/RXR binds to the PPAR response element, which is a specific DNA sequence in the promoter region of target genes, leading to the control of their expression. The PPAR response element is a hexanucleotide (AGGTCA) repeat separated by only a single nucleotide and varies for each PPAR member. All the members of PPARs are activated by the specific ligand concentrations (usually in µM range) both in the case of humans and ruminants [13].

A literature survey showed that information regarding the role of PPARs in lipid metabolism, the regulation of the expression of different genes and proteins and tissue distribution is mainly available in humans compared to dairy animals. However, Bionaz et al. analyzed the relative distribution of PPARs in bovine tissues of dairy cows and bovine cell lines through gene expression analysis by qPCR [14]. Their findings showed that the overall relative distribution of PPARs in dairy animals is quite similar to other species. Later, some studies also showed the relative distribution of PPARs in different organs of dairy animals, including rumen, adipose tissue, liver, kidney, lungs and mammary tissues. The biological and metabolic roles of PPARs have shown that they are the major molecules that regulate energy homeostasis [15], and hence, they are ideal candidates to address metabolic disorders in dairy animals through nutritional interventions.

## 2. Nuclear Receptors’ Mode of Action

**Figure 2:**
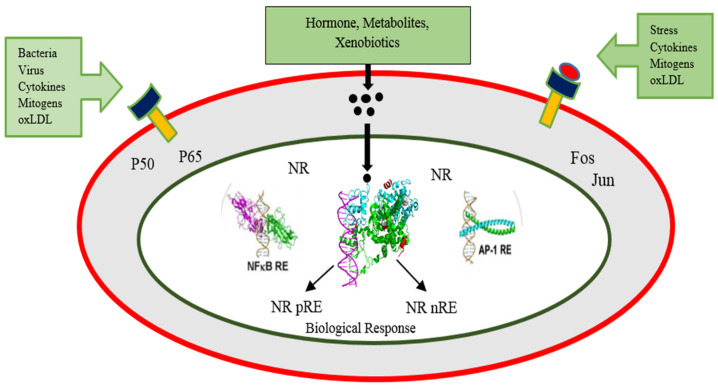
Nuclear receptors work in two apparent manners. Firstly, through the binding of a ligand, these receptors can frame, Heterodimers with RXR that outcomes in their connection with a specific positive response element of gene and, in this manner, can cause transcription of mRNA of genes that are targeted. On the other hand, repressive, negative response elements (nRE) have likewise been observed to interact with these receptors [9].

The potential mode of action of nuclear receptors is a prerequisite to better understanding the role of PPARs in energy homeostasis. The nuclear receptors can control transcriptional events by exerting a positive effect directly or by repressing regulated promoters. The protein–protein interactions can mediate a repressive effect on other signaling pathways under the regulation of transcription factors such as AP-1, NF-kappa-B, or C/EBP [9]. Figure 2 describes the involved elements and the processes that elicit the biological response.

### 2.1. Transcriptional Activation

Generally, transcription activation includes ligand-dependent conformational modifications of the chromatin REs-associated nuclear receptors, activating corepressor complex discharge and the successive deployment of coactivator complexes that alter the chromatin structure and facilitate the transcription initiation complex’s assembly at the promoter regions. Numerous NR coactivators have been identified, and the repertoire is unique to a few cell types, signals, and genes. Therefore, the agonists binding activates the transfer of corepressors for coactivators critical for the transcription activation process. Moreover, the ligand-dependent interaction of NR corepressors, such as LCoR and RIP140, through LXXLL motifs could hinder the transcription process [16,17].

### 2.2. Nuclear Receptor Corepressor Binding

In the absence of ligands, the NRs are found to be associated with the corepressor complexes. These complexes consist of a subunit (SMRT/NcoR2 or NCoR1) that interacts directly with the receptor by means of the LXXLL motif that has a consensus sequence (L/I-X-X-I/V-I or LXXXI/LXXXI/L) also referred to as the CoRNR [18,19]. This CoRNR box motif interacts, as the coactivator LXXLL motif, with amino acids from the LBD hydrophobic groove. Part of the CoR binding interface is obscured as the remodeling of the binding of agonist and helix 12 positioning takes place. The availability of CoR binding interfaces as well as new CoRNR boxes indicates the use of alternate methods for the interaction of NR–corepressor [20,21,22]. The corepressor complexes are also developed around the NCoR or SMRT subunits that have a conserved repression domain and act as a vital point for core repressive machinery (such as GPS2, HDAC3, TBL1/TBLR1B) to assemble. Under certain situations, ligand-binding is adequate to prevent the recruitment of corepressors, such as RXR and TR, but in these cases, the active corepressor complex needs to be eliminated.

### 2.3. Nuclear Receptor Coactivator Binding

The identification of SRC-1/NCoA1 as a coactivator of the progesterone receptor [23] led to the discovery of more than 350 coactivators up to now. This tremendous volume of polypeptides had their involvement in different enzymatic processes related to the chromatin remodeling, histone modulation, transcription initiation and elongation, mRNA splicing and elongation and nuclear receptor complexes’ proteasomal termination [24]. These coactivators are further categorized into two subfamilies. The members of the first subfamily of coactivators are involved in the direct interaction with NR AF-1 and 2 regions such as SRC coactivators, CBP and p300. The members of second subfamily coactivators can interact with primary coactivators such as CoCoA, CARM1 and Fli-I. The primary and secondary activators work in a coordinated manner to regulate the promoters [25].

#### p160 and p300 Families

Coactivators such as P160, p300 and cAMP-response element-binding protein (CBP) belongs to the p160 family such as NCoA1/SRC-1 and NCoA2/TIF2, commonly referred as SCRP1 or GRIP1, and possess binding affinity with the LBD of NR by means of an Alpha-Helical LXXLL motif [26]. The p160 family coactivators include NCOA 1/SRC-1, NCOA 2/TIF2 (SRC-2 or GRIP1) and NCoA3/RAC3, also known as SRC-3, ACTR, PCIP or TRAM-1. The p300 and CBP coactivators have a histone acetylase transferase (HAT) function, which plays a crucial role in NR-mediated transcriptional regulation [27]. The acetylation of histone H4 N-terminal tail inhibits the interactions of the histone H4 N-terminal with the histone H2A/H2B dimer and disrupts chromatin compaction [28]. Thus, the chromatin is then decondensed causing the initiation complex at the promoter site to be attached.

### 2.4. Transcriptional Repression

#### 2.4.1. Transcriptional Repression by Unliganded Receptors

In the absence of a ligand, some nuclear receptors can effectively downregulate the transcription. Thus, corepressor complexes recruiting is linked to this process. The most widely studied complex is the nuclear receptor corepressor (NCoR) that acts as a silencing mediator of thyroid and retinoid receptors (SMRT), G-protein pathway suppressor 2 (GPS2), histone deacetylase 3 (HDAC3), TBL-1-like related protein (TBLR1) and transducin-α-like 1 (TBL1). A well-characterized function of HDACs in transcriptional repression is to create a condensed, transcriptional inactive chromatin structure by the N-terminal lysine of histone proteins deacetylation. The SMRT and NCoR have been reported to possess a deacetylase-activating domain which can activate the enzymatic activity of HDAC3 [29].

Moreover, some other corepressor complexes, such as the SWI/SNF complex, CoRest and PRC1 and 2 complexes, have been further identified. Similarly, the NCoR/SMRT complex can bind with multiprotein elements of the promoter’s site, resulting in covalent histone and DNA changes, accompanied by chromatin contraction and/or DNA masking. The dissociation of the corepressor complex from the DNA-bound receptor is a key step in NR-mediated transcription activation. In vitro experiments along with the data from the crystal structures have shown that agonist-induced conformational changes are adequate for SMRT or NCoR alienation. However, dynamic models of de-repression involving post-translational modifications of corepressor complex subunits leading either to their nuclear exclusion and/or degradation have been described [30].

#### 2.4.2. Direct Trans-Repression by Ligand Activated Receptors

The negative transcription regulation of certain genes can be repressed by ligand-bounded NRs. The mechanistic role of these ligand-bounded NRs has already been described in-depth for the GRs and TRs. These NRs have been suggested to recognize, bind and downregulate particular target genes. Studies have shown that the response elements which negatively regulate the glucocorticoid or rnGREs and thyroid elements or nTREs differ from those of response elements that positively control the activation of transcription process [31,32]. The negative response elements for GR and TR possess the overlapping binding sites that control the response elements transcriptional cis-effect for transcriptional factors such as Oct-1/Pbx, AP-1 and SP1 [33,34,35]. This indicates that the negative glucocorticoid reaction elements associated with other transcription factors can exert such an action. A recent study has identified a new class of negative glucocorticoid REs, which are arranged as 1 bp spacers inverted repeats and facilitate the glucocorticoids to promote the recruitment of GR–corepressor complexes [36]. For T3-mediated repression of transcription, a similar type of mechanistic principle does not exist. The SMRT corepressor insertion in nTRE enhances the histone deacetylation that has also been reported for the αTSH gene. The SMRT dissociation is associated with histone acetylation and gene suppression after treatment with an agonist [32,37]. Additionally, functional studies have revealed the involvement of SRC-1 in liganded TR transcriptional repression [38,39]. The mechanism involved in the reversal of the transcriptional function is not clear yet, but it can be regulated by means of post-translation changes, including acetylation or SUMOylation of promoter-associated histones, phosphorylation and/coregulatory proteins [24,40]. Therefore, direct repression could happen through distinct receptors and context-dependent pathways. These findings also indicated the versatility of coregulator complexes that either positively or negatively impact the products of the transcription following the stimulation by NR agonists.

#### 2.4.3. Tethered Transrepression by Liganded Receptors

The process called the tethered transrepression contains negative crosstalks of ligand-activated nuclear receptors with other signal-dependent transcription factors, including NF-kappa-B and AP-1. Inflammation in different cells of the central nervous system, the immune system and in the liver, etc., is modulated by this process and also interferes with cell proliferation in many tissues. Various putative mechanisms have also been proposed to explain such repression: (i) the inhibition of PIC assembly on NF-kappa- or AP-1-regulated promoters; (ii) the inhibition of RNA polymerase II change to elongation-competent form; (iii) the upregulation of NK-kappa-B inhibitors [41]; (v) the coactivators exclusion by competitive inhibition [42,43]; (vi) direct physical interaction with AP-1 or NF-kappa-B subunits (p65 commonly) [43], but this mechanism is even more complicated and intricate with several other factors in the cell [44].

Moreover, after being partly affected by PPARγ, GR and LXR agonists, for each receptor, the inhibition was about one-third or half of the gene induced by TLR-3, 4 or 9 active macrophages inflammatory elements. Interestingly, each receptor was partly overlapping with inhibited clusters of genes [45].

The NR structural features unique to transrepression are not well described yet. Research using comprehensive mutagenesis of T3R, RAR, PPARμ, GR and ER has not provided a simple, harmonized model for tethered transrepression [46,47]. Thus, it is evident that the coactivators recruitment through the Domain AF-2, as well as direct DNA connections, is not necessary for this process. Furthermore, it also became apparent that homo- or heterodimerization was not obligatory [47,48]. The unavailability of defined molecular structures for transrepression is the major hindrance in devising screening methods for the detection of dissociated ligands that preferentially induce tethered transrepression in inflammatory diseases.

## 3. Role of PPARs and Coregulators in Energy Homeostasis

Energy is an absolute necessity to provide subsistence to all the living beings and is usually derived from the metabolism of ingested nutrients. Primarily, in human beings, glucose and long-chain fatty acids derived from food are utilized to produce energy. The cellular requirement for energy is satisfied through the oxidation reactions occurring in mitochondria to metabolize nutrients. Both in normal as well as in induced cells, the transcriptional regulation network controls the demand and supply of diverse cellular physiological states in both normal and induced cells such as during fasting or exercise. PPARs are classified as a part of the superfamily of nuclear receptors within this transcriptional network and control the nutrient-dependent transcription [49,50,51]. PPARs function as fatty acid sensors in order to control various metabolic processes, and different biological activities such as inflammation, adipogenesis, insulin sensitivity, lipid metabolism, reproduction as well as cell growth and differentiation. PPARs control these functions through the activation of target genes via the attachment of endogenous ligands to the receptors’ ligand-binding domain. This binding leads to conformational change, which further enables PPARs to heterodimerize the retinoid X receptor and facilitate the attachment and dissociation of transcription-related important small accessory molecules. This heterodimerized complex formed at the PPAR response elements (PPREs) then transactivates target mitochondrial and peroxisome-related genes. This event cascade controls a protein network concerned with systemic energy homeostasis [51,52,53]. All the isotypes of PPARs have an indispensable role in lipid and fatty acid metabolism through direct binding or the modulation of the target genes related to fat metabolism [49]. Though all these PPAR isoforms share similar mode of action and function, distinct biological and pharmacological differences exist among them. The PPARβ/δ and PPARα have a metabolic role in the promotion of energy dissipation, but PPARγ stimulates the energy storage. PPARβ/δ improves fatty acids oxidation in different body tissues and also normalizes plasma lipid content. PPARβ/δ and PPARγ boost insulin sensitivity, while PPARα do not. PPARβ/δ-mediated glucose regulation is different from that of PPARγ; however, PPARβ/δ and PPARγ both are implicated in fiber distribution in the skeletal muscle, metabolism of glucose in the liver and controlling pancreatic islet cells function [53]. In lipid catabolism, the PPARα enhances the fatty acid oxidation during situations such as fasting, while PPARγ acts on the adipose tissue during the anabolic process to improve lipogenesis [54,55]. Thus, understanding the role of PPARs in energy homeostasis is important to further investigate the PPARs’ role in producing energy in different body parts.

### 3.1. The ATP-Dependent Remodeling Complex SWI/SNF

In yeast, the SWI/SNF complex is an evolutionarily conserved multi-subunit complex, which uses the energy of ATP hydrolysis to mobilize nucleosomes and remodel chromatin and thereby regulate the transcription of target genes. In ATP-dependent chromatin restructuring [56], the evolutionary conserved SWI/SNF families play a significant role in catalyzing the DNA histone disruption and the nucleosome sliding around the DNA [57]. A multimeric agent of 1.2 MDa is the human homolog BAF complex of BRG1/hBRM, BAF Polypeptides (BAF45a/b/c/d, BAF47, BAF53a/b, BAF57, BAF155/170, BAF60, BAF250a, BAF200, Brd7 and Brd9) and actin. A number of these subunits have LXXLL motifs which have not only been identified in the form of ER, RR [58], RAR [59], FXR [60] and GR [61] coactivators but also as SHP corepressors [62], which also incorporated with corepressor complexes in order to combine SWI/SNF components [63]. Intriguingly, in the mouse liver, the BAF 60a subunit showed a circadian expression, which regulates the expression of clock and metabolic genes by acting as a coregulator of RORα [64].

### 3.2. The Mediator Complexes

The mediator complex was initially identified in yeast, such as the SWI/SNF complex, and consequently characterized in many other eukaryotic cells. Many studies have identified its role as a catalyst for the transcription pre-initiation complex, abbreviated as PIC, assembly of activated promoters. The mediator plays a major role in RNA II-controlled transcription mechanism by direct association with RNA polymerase II, such as TFIID and TFIIH, and elongation factors [65]. Different studies have reported the function of the mediator in NR and made it clear that complexes linked to mediators are specifically associated with NRs. The mediator consisted of four structural modules and had more than 20 subunits, among which LXXLL motifs [66] were developed from the Med1 subunit. The hepatic steatosis Med1 KO causes PPARα-dependent steatosis [67], in line with the coactivator functions of the liver [68] and PPARα [69].

### 3.3. PPARs Signaling in Different Body Parts

The liver is the prime body organ involved in energy metabolism to fulfill the body’s energy requirements, and PPARα receptors are also distributed in the liver, which controls the uptake and breakdown of fatty acids through ketogenesis and β-oxidation in fasting conditions [70,71]. It has also been described that PPARα knockout in mice causes the suppression of fatty acids uptake and oxidation and the impairment of ketogenesis as well as gluconeogenesis. Furthermore, the function related evidence for PPARβ/δ were also reported as the PPARβ/δ knockout decreases the expression of genes involved in glucose and lipogenesis, while PPARβ/δ overexpression controls genes that are responsible for energy metabolism [49]. In the liver, PPARα is indispensable for glucose homeostasis. The mice deficient in PPARα showed a substantial blood glucose level reduction following 24 h of fasting. The upregulated expression of TRB3 (a positive controller of the cellular reaction to the levels of insulin and Akt/protein kinase B blocker) directly regulates the PPARα transcription that negatively influences insulin signaling [72]. Moreover, PPARα also enhances the production of acetyl-CoA enzyme and fatty acids oxidation through upregulating Acyl-CoA dehydrogenase expression in mitochondria. PPARα controls de novo lipogenesis in the case of positive energy balance to provide fatty acids, deposited in the form of triglycerides, which can also be employed during starvation [73].

Brown adipose tissue (BAT) acts as a caloric storage site, and white adipose tissue (WAT) as lipid storage also holds importance in energy homeostasis. These tissues are involved in endocrinal functions, which also release different types of hormones, including adipokines and cytokines, which subsequently initiate systemic energy metabolism signaling. Through feedback mechanisms, these tissues control energy homeostasis by receiving signals from the metabolic active sites in peripheral tissues and the central nervous system [74,75,76]. Substantially, PPARγ is expressed in these tissues and plays a lead role in the gene activation required for the uptake and deposition of fatty acids as well as the differentiation of adipose tissue [77]. Non-adipogenic cells are differentiated into adipocytes through the ectopic expression of PPARγ [78]. The PPARγ knockout in embryonic fibroblasts completely disrupts the differentiation process [79]. In vivo studies have revealed the importance of PPARγ for adipocytes production and survival in animals as negative mutations (heterozygous and dominant) in the PPARγ in humans cause lipodystrophy [15,80]. In BAT, PPARα controls the expression of mitochondrial uncoupling protein 1 (UCP1) and PGC1α, but the obliteration of PPARα decreases the protein expression upon exposure to normal and cold conditions while the fatty acids’ metabolism is not affected. The enhanced energy metabolism has also been observed in response to the enhanced expression of the FAO gene induced by the activation of PPARα in human and murine adipocytes [49]. Liu et al. reported PPARγ as a positive regulator of milk fat synthesis in dairy cow mammary epithelial cells through improving cell viability, proliferation ability and triacylglycerol secretion [81]. It was also reported that acetic acid and palmitic acid could regulate milk fat synthesis in dairy cow mammary epithelial cells through PPARγ signaling. Shi et al. have cloned the PPARγ gene in the dairy goat mammary gland and explored its function in vitro [82]. It was reported that PPARγ in the goat mammary gland directly controls the synthesis of milk fat through the activation of the transcription regulators, such as sterol regulatory element-binding transcription factor-1 [82,83].

Skeletal body muscles are the significant sites for glucose usage mediated through insulin, lipids metabolism, glycogen storage and oxidation of fatty acid as well as regulation of HDL and cholesterol levels. PPARβ/δ expression is dominant in the skeletal muscles and controls the translation of genes associated with energy metabolism [71,84,85,86]. Moreover, it also regulates the activity of genes related to triglyceride hydrolysis, lipids uptake, fatty acids oxidation, and uncoupling proteins activation to liberate the energy required by OXPHOS. The protein CPT1 is also programmed to regulate the oxidation of the long-chain fatty acids. PPARβ/T activates the metabolic adaptability of the transcription factor FOXO1 and the pyruvate dehydrogenases kinase 4 (PDK4), which inhibits the complex of pyruvate dehydrogenase. This makes CPT1 a rate-limiting factor for the oxidation of carbohydrates in the muscles. Moreover, PDK4 also controls the regulation of several genes that are involved in lipid efflux, energy usage and increases β-oxidation of fatty acids [84,85]. Furthermore, in PPARβ/δ transgenic mice, metabolism of glucose was greatly amplified [84] as PPARβ could initiate the transcription of lactate dehydrogenase B (LDHB) to regulate the muscle fatty acid metabolism required for glucose oxidation [87]. On the other hand, PPARγ coactivator-1α or PGC-1α, which is a mitochondrial biogenesis regulator, controls the energy metabolism in skeletal muscle through catabolic reactions to produce aerobic ATP. The PPARβ/δ stimulates the expression of PGC-1α to control the skeletal muscles’ metabolic activity by enhancing the synthesis of mitochondrial proteins [88,89,90].

The PPARα and PPARβ/δ are predominantly expressed in the intestines [91,92], and the triglycerides’ metabolism in the intestine is crucial for systemic energy homeostasis. Dietary triglycerides are hydrolyzed into free fatty acids in the intestinal lumen and then taken up by epithelial cells of the intestine to the endoplasmic reticulum and again converted into triglycerides [92]. Studies in animals have shown a relationship between energy utilization, intestinal colonization and weight gain that controls the angiopoietin-like protein 4 (ANGPTL4) expression in the intestinal epithelium. ANGPTL4 is a secreted protein that regulates lipid and glucose homeostasis, and its deletion leads to changes in metabolism, reduced oils absorption in the intestine and intestinal mucosa thickening. PPARγ is reported to be involved in the regulation of the fatty acid metabolism via β-oxidation. PPARγ controls ANGPTL4 expression, and short-chain fatty acids stimulate PPARγ and are the major energy resources for colonocytes [93]. Wy-14643 is a PPARα agonist that stimulates the production of the enzymes implicated in fatty acid oxidation and ketogenesis, such as mitochondrial 3-hydroxy-3-methylglutaryl-CoA synthase and CPT1A in the small intestine [94]. PPARα also controls different phase I enzymes and transporters (related to oxidation) as well as uptake of fatty acids. PPARα is activated through the nutritional route and regulates fatty acid oxidation, cholesterol and glucose transporters [95]. PPARα is crucially involved in the regulation of the phage I/II metabolism and also controls the expression of transporter genes in the small intestine [96]. A synthetic agonist (K-877) of PPARα has been shown to control the intestinal fatty acid oxidation and mRNA expression of apo-lipoprotein while reducing plasma levels of triglycerides. The downregulation of Npc1l1 and upregulated expression of Abca1 has been observed in response to treatment with K-877. Npc1l1 is a rate-restricting transporter for absorption of cholesterol in the murine small intestine, while Abca1 is a vital molecule that participates in the production of HDL-C in the small intestine [97].

### 3.4. Energy Homeostasis through Co-Regulators of PPARs

The energy homeostasis could be controlled through feedback mechanisms involving various types of extraordinarily interconnected pathways. About 320 coregulators and 38 co-modulators for PPARs have been reported in the Nuclear Receptor Signaling Atlas. The direct interaction of PPARs and the crosstalk of PPARs with other pathways contribute to systemic energy homeostasis [98].

Balanced mitochondrial energy production is being regulated through the coordinated effect of coactivators and corepressors where PGC-1α and PPARγ act as co-modulators for the initiation of mitochondrial aerobic metabolism. However, the effect of PGC1α on mitochondria is antagonized via nuclear corepressor 1 (NCOR1). The knocking out of NCOR1 has been shown to imitate the overexpression of PGC-1α phenotypically, which is involved in the transcriptional output of ERR and PPARs. Nuclear receptor interacting protein 1 (NRIP1) interacts with both PPARs and ERR and decreases the target gene expression levels that participate in the consumption of energy. In previous studies, the mice with the deletion of NIRP were slim and presented enhanced insulin sensitivity and glucose tolerance and endurance to diet-induced obesity [50,98,99]. Hes6 protein, hepatocyte nuclear factor α (HNFα) and the PPARs coregulate each other’s expression under different nutritional conditions and also control the transcription events during the metabolic reactions [80,100]. PPARγ, along with the transcription factor such as CCAAT/enhancer-binding protein α (C/EBPα), is a crucial regulator in the last phase of adipogenesis. Energy homeostasis by the mediator complex subunit 1 (MED1) through PPARs plays an essential role in a liver-specific knockout of MED1 and demonstrated impaired activities of PPARα and PPARγ in murine models [98].

Since the role of PPARs in different energy homeostasis cascades in various organs has been established, it can be stated that PPARs could be the target for the treatment of disorders, such as inflammation, obesity, diabetes, dyslipidemia, neurodegenerative disorders and cardiac myopathy, when these cascades are disrupted in disease conditions due to metabolic energy imbalance [15,101].

## 4. Nutritional Modulation of PPARs to Modify Gene Expression and Metabolic Networks

Dietary nutrients can modulate the metabolic networks of PPARs as nutrients, and their products directly control the PPAR activities through acting as natural ligands of PPARs. Diverse nutrients have been shown to affect the action of PPARs, but PPARs depict the greatest inclination for mono-unsaturated and poly-unsaturated fatty acids as demonstrated by different ligand-binding assays [102,103]. It is evident that each type of PPAR triggers a distinct gene network regardless of their overlapping expressions, which indicates the exhibition of ligand-specific properties of PPARs [103,104]. Furthermore, the administration of high-fat diet results in the modulation of PPARα target genes [105]. Comparative nutrigenomic study in mice revealed the influence of several individual dietary fatty acids on hepatic gene expression. These findings concluded that (1) an increase in the chain length of fatty acids and the extent of unsaturation enhanced the total genes that were upregulated and that (2) genes controlled through dietary unsaturated fatty acids do not change in the PPARα knockout murine model depicting PPARα as end target, and the expression levels of same genes were increased in the murine model after the administration with the PPARα activator WY14643 [106].

The modulation of PPAR expression and function through nutrients can be studied by imposing nutrient deprivation conditions on diverse tissues. The properties of all known PPARs are influenced in the fasting state; for example, PPARα signaling in the liver has shown to be upregulated via fasting through increased expression levels of the coactivator PGC-1α, and thus controls hepatic gluconeogenesis and fatty acid oxidation [107,108]. Furthermore, increased expression levels of PPARδ during fasting are affected by plasma fatty acids derived from adipose, hence highlighting a distinct task as a plasma fatty acid sensor in the liver for PPARδ [108].

**Figure 3:**
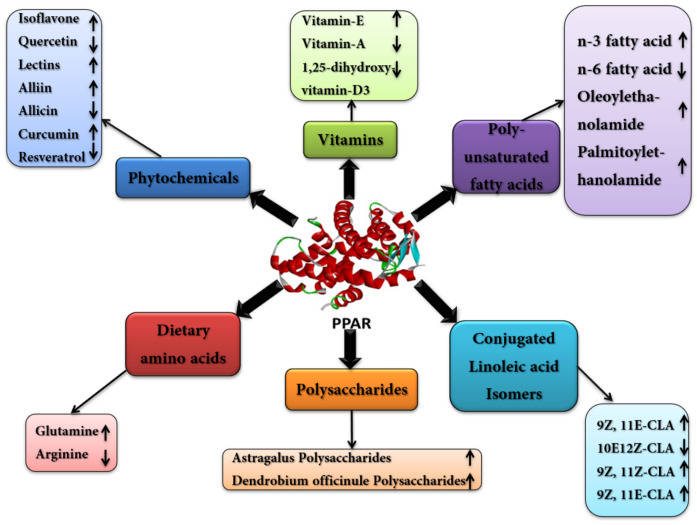
The effect of different nutrients on PPAR. Different nutrients regulate PPAR either by its upregulation or downregulation. The arrow up shows the upregulation of PPAR, while the arrow down shows the downregulation by respective nutrients.

Several nutrients and their derivatives are being observed for the modulation of PPARs to modify metabolic networks and gene expression through direct and indirect mechanisms. Macronutrients such as nucleotides, fatty acids and their metabolites, amino acids, monosaccharides and micronutrients, such as vitamins, can control the expression of specific genes directly by interacting with transcription factors in the promoter region through cis-regulatory elements. However, many nutrients regulate genes indirectly by modulating the intracellular action/secretion of hormones, such as thyroid hormone, glucocorticoids, glucagon and insulin, which alters the gene expression and thus improves metabolic networks. Many dietary nutrients have been shown to modulate the expression of PPARs in animals (Figure 3), among which some significant factors are described below.

### 4.1. Poly Unsaturated Fatty Acids (PUFA)

Polyunsaturated fatty acids are categorized as *n-3* and *n-6* fatty acids and could exert opposing effects on receptor signaling. Out of these two classes, *n-3* fatty acids are shown to have an agonistic effect, while *n-6* fatty acids are reported to be inhibitory [109]. PUFAs are shown to bind directly to the PPAR*α* and are involved in the activation of transcription, thus controlling metabolic networks. It has been reported that PUFAs are required in the μM range to bind with PPAR*α*, and these could be derived from dietary nutrients [110]. Interestingly, *n-3* fatty acids are reported to be greater activators of PPARα as compared to *n-6* fatty acids in vivo [111]. Furthermore, many eicosanoids and their derivatives are shown to activate PPARα with a high affinity than other PUFA precursors [112]. Studies have represented that acylethanolamines, including oleoylethanolamide (OEA), palmitoylethanolamide (PEA) and anandamide (AEA) are also PPAR*α* activators [113]. Moreover, PPARα activation by oleoylethanolamide (OEA) leads to appetite and lipolysis suppression, while palmitoylethanolamide (PEA) exerts anti-inflammatory activity when activating the PPARα [114]. The ligands for PPAR*α* are also known to bind PPARβ/δ, but their activation is lower than the PPARα. PUFAs also serve as ligands for PPARγ and are involved in the activation of PPARγ. For example, n-3 fatty acid activates the PPARγ and can result in the prevention of high-fat-diet-induced inflammation in adipose tissues [115]. Collectively, PUFAs are the natural ligands for all the subtypes of PPARs, but their subsequent activation potential varies. These molecules control the PPARs activity in the body and thus have a role in regulating metabolic networks. Although various studies have reported their mechanism of action to activate PPARs, further research is still needed to elucidate the mechanisms of PPARs activation and their distribution.

### 4.2. Conjugated Linoleic-Acids (CLAs)

CLAs are the fatty acids mainly found in foods obtained from ruminant animals [116] and are positional (*cis*- or *trans*-double bond positioning at 7, 9; 8, 10; 9, 11; 10, 12; or 11, 13) and geometrical isomers of the parent linoleic acid molecule (*cis*-9, *cis*-12-18:2, *n*-6). Rumenic acid (9*Z*, 11*E*-octadecenoic acid, C18:2) is the most abundant natural CLA isomer (over 75–80%) produced through the biohydrogenation of nutritive LAs by ruminant microflora. Because of their numerous health benefits, CLAs are currently being used as nutritional supplements for changing body composition in livestock and humans [117,118], but the mechanisms of the useful properties of CLAs are yet to be explored. CLA isomers serve as ligands for PPARγ, PPARβ/δ and PPARα [119,120], showing differential PPAR activation and health benefits [118,121] (Table 2).

Additionally, a mixture of CLA isomers, i.e., 9Z, 11Z-CLA and 9Z, 11E-CLA, can notably activate the PPARβ/δ in preadipocytes [122]. Therefore, minor structural changes in many CLA isomers can be differentiated by important cellular mechanisms to allow specie and tissue-specific responses [123]. These findings concluded that CLA affects the production of eicosanoids directly or indirectly, abolishes the NF-κB pathway, improvises the activation of PPARγ and decreases proinflammatory cytokines for useful effects on inflammation, ultimately manipulating metabolic syndrome-related conditions, including IR, atherosclerosis and obesity [124]. Therefore, CLAs can directly employ anti-inflammatory properties by modulating the expression of inflammatory mediators through PPARγ-dependent or NF-κB-dependent pathways.

### 4.3. Dietary Amino Acids

Some of the dietary amino acids have shown the potential to modulate the activity of PPARs, in which glutamine and arginine are the major ones. Glutamine is considered an essential amino acid in situations of metabolic stress and is found to be a special substrate of enterocytes. To date, only a single study has reported the impact of glutamine on PPARγ articulation. Sato et al. examined the impacts of luminal glutamine and arginine on the activity of PPARγ in gut ischemia-reperfusion of a rat model. Luminal glutamine increased the expression of PPARγ, while arginine did not show any significant effect on PPARγ. Furthermore, they also evaluated the effect of a PPARγ antagonist (GW9662) on the action of glutamine. The pre-treatment with GW9662 revokes the impact of glutamine, revealing that glutamine may likewise be a PPARγ agonist, thus signifying its role in metabolic stress [125].

Moreover, the effect of arginine on a gut injury has been investigated, and the supplementation of arginine, which is considered an immune-nutrient, demonstrated a beneficial effect on LPS-induced gut injuries in a pig model [81]. Additionally, upon treatment with arginine, there was a decrease in jejunal TNFa, and an increase in the expression of PPARγ was also observed.

### 4.4. Vitamins and Minerals

#### 4.4.1. Beta Carotene, Vitamin A, and Its Derivatives

In mammals, beta carotene (BC) is the precursor of apo-carotenoid molecules, i.e., retinoids (vitamin A and its derivatives) [126]. There is an increasing sign that BC and retinoids can affect the physiology of adipocytes as signaling molecules by acting on adiposity in humans [127]. The levels of circulating BC are inversely associated with the risk of human type-2 diabetes [128,129,130], while the decreased levels of plasma carotenoids, including BC, are usually found in obese children [131].

The BC 15,15′-monooxygenase (*Bcmo1*) is the major contributing enzyme for the production of retinoid, which converts BC into all-*trans*-retinal [132]. Its expression is controlled by PPAR-γ [133,134] induced during the differentiation of adipocyte [135], and *Bcmo1* knockout mice showed an enhanced expression of PPAR-γ genes in fat-deposits and are very susceptible to fat-induced obesity [132]. Retinaldehyde, the primary product of BC cleavage, inhibits the activity of PPAR-γ both in mouse models and adipocyte cell cultures [136]. The role of *Bcmo1* is verified in signaling of the RA receptor (RAR) and the production of Retinoic acid (RA) in adipocytes [135]. Furthermore, BC-derived long-chain apo-carotenoids, such as β-13-apocarotenone, proved to inhibit the activity of retinoid X receptor-alpha (RXRα), while β-apo-149-carotenal hinders the adipogenesis and activity of PPAR-γ in cell culture [137]. BC supplementation can reduce the activity of PPAR-γ and downregulate its target genes, decreasing the adiposity of mice. Thus, BC can significantly control the adiposity in mice, and *Bcmo1* critically regulates the PPAR-γ, which is the key element for the connection between PPAR-γ and RAR-signaling pathways that ultimately control the body adiposity [138].

#### 4.4.2. Vitamin E: Alpha Tocopherols and Tocotrienols

Vitamin E is the fat-soluble vitamin family comprised of 8-lipophilic natural compounds including four tocotrienols with an unsaturated-isoprenoid sidechain designated as α, β, γ, and δ, and four tocopherols with a saturated phytyl-tail [139,140]. Soybean, cottonseed and corn are the commercially produced vegetable oils that have high amounts of most common dietary tocopherols (α- and γ-tocopherols) [141,142]. Both α- and γ-tocopherol shown to activate expression of PPAR-*γ* and transactivation of cancer cells in the colon, but *α*-tocopherol modulate PPAR-γ expression better than *γ*-tocopherol [143,144].

Tocotrienols are non-toxic naturally occurring compounds used as dietary supplements to prevent damage with aging due to dysregulated inflammatory responses. Recently, in vivo anti-inflammatory properties of dietary supplements evaluated in mice and chickens with two natural proteasome-inhibitors, i.e., δ-tocotrienol and quercetin [145,146], revealed decreased levels of nitric oxide [147] and serum tumor necrosis factor-alpha (TNF-α). Furthermore, the direct effect of tocotrienols on lipidic metabolism, with an anti-atherogenic effect on rats, humans and mice, has been also reported.

In vitro studies revealed that tocotrienols inhibit the 3-hydroxy-3-methyl-glutaryl-CoA reductase and consequently decrease cholesterol synthesis. For instance, the body fats in rats were decreased by the oral administration of a tocotrienol-rich fraction (TRF) of palm oil containing *γ*-tocotrienol, while in an in vitro study, the phosphorylation of Akt in 3T3-L1 preadipocytes and adipocyte differentiation was suppressed by TRF through the reduced expression of insulin-induced PPAR-γ [148]. Tocotrienol can serve as an anti-adipogenic vitamin due to nutrient-mediated regulation of body fat, but further research is required in this regard.

#### 4.4.3. Retinoic Acid and 1,25-Dihydroxy Vitamin D3 (1,25(OH)_2_D_3_)

Some of the properties of RA, such as the deposition of fats [149], adipocyte differentiation [150,151] and the expression of adipokines, such as resistin, leptin and serum retinol binding protein, is facilitated by RAR, which interferes with the activity of PPAR-γ [149,151]. The 1,25-dihydroxy vitamin D3 (1,25 (OH)2 D3) is an active form of vitamin D and has been shown to restrict the adipogenesis in the bone marrow of SAM-P/6 mice associated with decreased expression of PPAR-γ2 [152]. The suppressed expression of PPAR-γ2 by RA and 1,25-dihydroxy vitamin D3 inhibit the differentiation of adipocytes in 3T3-L1 preadipocytes [153].

### 4.5. Phytochemicals

Plants possess biological active chemical compounds that are known as phytochemicals [162]. Importantly, flavonoids, lectins, alliin, allicin, curcumin, triterpenes and resveratrol have been observed to regulate lipid and glucose metabolism through the modulation of PPAR [127].

A soy isoflavone, genistein, regulates lipid metabolism by activating the PPAR-*γ* or PPAR-γ-independent mechanism [156]. On the contrary, quercetin is a flavonol that inhibits the activity of all isoforms of PPAR except PPAR-γ and prevents fat accumulation in the liver [119]. In literature, chicken feed supplemented with quercetin has prevented dysregulation of inflammatory responses by downregulating NO and TNF-α during aging, while isoflavones have the potential to induce cancer via hormone-dependent regulation [154].

The bioactivity of lectins from vegan sources has been reported several times related to immune responses or gastrointestinal tract during allergens exposure. Moreover, its adipogenic effect has also been reported in humans and animal tissues [155]. Banana, garlic and dietary lectins boosted the hematopoietic stem progenitor cell pool in addition to an adipogenic effect on mesenchymal cells of mice by enhancing PPAR-γ2 expression. Furthermore, these dietary lectins interact with insulin receptors and activate the Mitogen-activated protein kinase (MEK)-dependent Extracellular signal-regulated kinase (ERK) pathway [163].

For more than 5000 years, garlic has been used as a culinary spice and medicinal herb. It has abundant antioxidant and organosulfur compounds that impart antibacterial and anti-infectious properties to it. Alliin and allicin are organosulfurated compounds extracted from garlic that possess a cardioprotective effect and anti-inflammatory effect, respectively [157]. Alliin lowers the TNF-α serum level in humans while allicin lowers or inhibits the expression of the CCAAT-enhancer-binding protein, CCAAT-enhancer-binding protein-alpha (C/EBP)-α and PPAR-γ2 during human preadipocytes differentiation [158].

Curcumin is the principal component of turmeric with potent anti-inflammatory, anti-cancerous, and antioxidant activities. Curcumin can suppress sepsis through PPAR-γ and decrease IFN-γ production in primed lymphocytes and *iNOS* gene expression in infected macrophages [159]. On the other hand, resveratrol, which has strong antioxidant properties, along with anti-obesity, anti-carcinogenic, neuroprotective, anti-aging, anti-diabetic and analgesic activity, targets PPAR-γ [164]. Resveratrol modulates white adipose-tissue metabolism and prevents dysregulation of advanced glycosylation end-products (AGE) via PPAR-*γ* mediated suppression of receptor for AGE in macrophage. It upregulates SIRT1, FOXO1 and adiponectin and downregulates PPAR-γ1−3 mRNA expression in human visceral adipocytes [160].

Polysaccharides and triterpenes have been used as a treatment for atherosclerosis and inhibit invasive behavior, angiogenesis and proliferation in cancer models. Moreover, they significantly promote adiponectin production and adipocyte differentiation by the downregulation of PPAR-γ, SREBP-1c and C/EBP and suppress the expression of genes involved in lipid transport, synthesis and storage [161].

## 5. Biological Benefits of PPARs Modulation in Dairy Animals

Overall, the comparison of the function of PPARs in humans, mice and ruminants has revealed that PPAR isotypes have a similar role in the metabolism of lipids in different species, including dairy animals. However, some differences are found in their specific roles across species as PPARs are found to be more specific for unsaturated FAs in monogastric species, while in ruminants, the PPARs are more specific for saturated long-chain FAs [165]. Aside from their role in the metabolism of lipids, the PPARs also influence immune responses through the modulation of immune signaling pathways such as AP-1, STAT-1 and NFkb through protein–protein DNA-independent interactions, a greater yield of milk production and controlling metabolic stress in dairy animals [166].

### 5.1. Energy Metabolism and Lipid Oxidation in Various Organs

PPARα is known to have an important role in fatty acid catabolism in mitochondria, peroxisomes and microsomes in the liver. PPARs are also believed to have a role in maintaining energy balance in various animals, such as ruminants and goats. Among its energy metabolism roles, carnitine homeostasis is well established in a diverse range of mammalian species, such as chicken, humans, mice, pigs, rats and diary animals [165]. Aside from PPARα, a few studies on the role of PPARβ/δ in energy metabolism have also been conducted. However, one role that is continuously associated with it is lipid metabolism. In the mammalian skeletal and heart muscles, the PPARβ/δ regulates fatty acid catabolism. These PPAR isotypes have also been found to have a crucial role in all of the reproductive tissues that have been investigated so far. In addition to this, research shows that PPARβ/δ has an important function in the metabolism of lipids in goat mammary cells, specifically lipid oxidation and secretion [83].

### 5.2. Adipogenesis and Milk Production

In ovines and bovines, PPARγ performs a critical role in adipogenesis, and its expression in the adipose tissues of these animals is high. PPARγ appears to be the critical mediator of lipogenesis by reacting quickly to stimuli signals in response to dietary energy intake [165]. For adipogenesis, PPARγ expression is both a requirement and a necessity. In bovines, PPARγ activation in bovine adipose tissue results in the upregulation of the genes that permit pre-adipocytes to differentiate into mature adipocytes or cells capable of storing triacylglycerol (TAG). One of the significant roles it plays in dairy animals from an economic benefit point of view is controlling the synthesis of milk fat in bovines. The PPAR expression increases during the transition state, which prevents the animal from metabolic stress [167]. The PPARγ gene is conserved in bovines, goats, humans and mice, as revealed by homology alignments. The research by Shi et al. confirmed the importance of PPARγ in modulating milk fat production. These findings showed that PPARγ is involved in regulating TAG production and release in the mammary cells of goats, highlighting the PPARγ’s functional significance of milk production in mammary cells. PPARγ affects the expression of genes involved in the regulation of milk fat in primary goat mammary epithelial cells as revealed by using a combination of experimental approaches, including gene expression analysis, PPARγ-specific activation, luciferase-PPRE tests and siRNA interference [82].

### 5.3. Controlling Inflammation

In dairy animals, PPARs activation is also associated with anti-inflammatory effects in dairy cows during their transition period [168]. A Japanese group showed for the first time that PPARγ could perform an anti-inflammatory effect in dairy animals by injecting human recombinant TNF with agonist Thiazolidinedione TZD into dairy steers for 9 days. They discovered that TZD therapy partially restored TNF-induced insulin resistance [169]. The TZD impact was most likely due to increased insulin signaling via PPARs activation, which also counteracted TNF [170]. In dairy animals, the anti-inflammatory impact of PPAR is evoked not just by mitigating the effects of TNF but also by lowering TNF synthesis. Bovine peripheral blood mononuclear cells treated in vitro with 100 M of conjugated linoleic acid isomer (t10, c12-CLA) inhibited the TNF production, thus controlling the inflammation and overexpression of pro-inflammatory cytokines [171]. These pieces of evidence highlight the importance of PPARs modulated through their agonists and antagonists to control inflammatory stress in dairy animals and can be further used as targets to reduce metabolic stresses.

### 5.4. PPARs and Fatty Liver Syndrome of Dairy Animals

Since PPARs bind to and are activated by long-chain fatty acids and their metabolites, the PPARs play an extremely important role in nutrient metabolism. Due to the industrialization of the dairy industry and extensive farming that aims to maximize profits from each dairy animal, milk and meat production of dairy animals, such as cattle and bovines, has increased. Among many factors, one such factor is the use of energy-rich diets containing high amounts of carbohydrates and lipids. However, the intake of such high caloric diets in combination with the sedentary rearing system in the industrial farms, diseases such as non-alcoholic fatty liver (NAFLD) have become common, which has substantially increased the morbidity and mortality rates of these animals around the world. Among various NAFLD conditions, fatty liver syndrome constitutes one of the most common disorders that affect dairy cows and buffaloes during the perinatal period, and it is triggered by a negative nutritional balance following calving. Dairy cows are a good model for studying pathologies such as fatty liver syndrome and various other NAFLD types and their etiology [172]. A recent piece of research revealed that fatty liver disease in dairy cows during early lactation is linked to poor hepatic mitochondrial activity (like abnormal acetylation of amino acid lysine). PPARγ has a role in NAFLD through regulation of glucose and lipid metabolism and differentiation of adipocytes as well as modulation of inflammatory responses in the liver. PPARγ controls the expression of various target genes in adipocytes, participates in the adipocyte differentiation, influences lipid metabolism and principally controls signal transduction in the pancreatic islet cells, all of which contribute to the onset and progression of NAFLD. PPARγ is important in modulating lipid oxidation and lipogenesis, in addition to its role in adipocyte development [173].

### 5.5. PPARs Interaction with Gut Microbiome and Animal Health

There is a dearth of information related to the role of PPARs and gut microbiome in dairy animals and how their dysregulation leads to various pathologies related to metabolic stress. A recent review by Hassan et al. has provided interesting information regarding the interaction between gut microbiome and PPARs and their overall effect on the health of humans. As high-calorie diets containing high carbohydrates diminish good bacteria that aid in metabolizing various nutrients, low-energy diets containing high fiber do the opposite. It increases the population of certain bacteria such as *Bifidobacterium, Lactococcus* and *Streptococcus* that have been shown to alleviate fatty liver syndrome. This is attributed to the increase in the population of folate-producing bacteria whose metabolic product, folate, induces PPARα, which is involved in lipid oxidation in the liver [174]. Since isotypes such as PPARγ are highly conserved among mammals, some of the information should be implicated in dairy animals to improve their economic output and health.

One of the effects of high-calorie diets in all mammals is the modulation of gut microbiota. High-calorie intake is thought to affect gut microbial balance via the TLR4–PPAR pathway. This can lead to (1) a rise in the number of microorganisms that produce inflammasomes (lipopolysaccharides) and (2) a reduction in the short-chain FAs. As a result, systemic inflammation rises, whereas the synthesis of short-chain FAs falls. Short-chain FAs activate PPARs in adipose tissue to control lipolytic genes, such as adipose triglyceride lipase and hormone-sensitive lipase, as well as lipogenic genes such as glycerol kinase and phosphoenolpyruvate carboxykinase, which aid in the appropriate metabolization and utilization of lipids. PPARs activity, on the other hand, declines as a result of a shortage of short-chain fatty acids, resulting in the formation of extra fat and subsequently its storage and increased inflammation [175].

### 5.6. Other Benefits

Extra-hepatic signals, including hepatokines such as fibroblast growth factor 21 (FGF21) and angiopoietin-like 4 (ANGPTL4) [176,177], have been described in monogastrics as PPARs targets that perform a key role in bovines related to the adaptation of tissues to low-energy state levels of the body, such as undernutrition, fasting and transition to lactation [178,179].

The downregulation of the glucose transport mechanism in the bovine endothelial cells caused by excessive glucose has been also shown to be regulated by PPAR/δ [180]. It has been previously demonstrated that activated PPARδ suppresses the solute carrier family 2 member 1 (or facilitated glucose transporter GLUT1) expression while, at the same time, increasing the calreticulin expression, a protein that promotes GLUT1 mRNA degradation. Given the low levels of circulating glucose in ruminants (<4 mM in dairy cows) [181] against ca. 5 mM in humans and >6 mM in mice), the condition investigated in the study (high glucose) has presumably minimal significance for ruminants. However, GLUT1 is among the most significant glucose transporters whose expression rises dramatically during the lactation period in mammary tissue of dairy cows [182]. Moreover, the modulation of glucose transport by PPARβ/δ might have ramifications in milk synthesis. As a result, these PPARs isotypes could be crucial in providing glucose for lactose production. Moreover, PPAR β/δ expression is significantly decreased in the mammary glands during lactation [183], which coincides with an upsurge in the expression of numerous glucose transporters, particularly GLUT1. If it is true, it opens up the possibility of employing PPARβ/δ antagonists to boost milk production.

Since PPARs have been identified as promising targets for improving metabolism and general wellbeing through nutritional interventions and various agonists and antagonists, further investigations are essentially required to provide physiological insights into the therapeutic role of PPARs in addressing various metabolic disorders in dairy animals.

## 6. Conclusions

PPARs are considered to be major nuclear receptors to control energy homeostasis in the body through various mechanisms in different body parts. Various nutrients can act as a ligand for PPARs for their modulation, in which PUFAs, dietary amino acids, vitamins and phytochemicals are the major ones. These nutrients modulate PPARs by regulating their expression and signaling in different body parts and lead to the control of metabolic networks.

## Figures and Tables

**Table 1:** Nuclear receptors along with their ligands [8].

Receptor Name	Abbreviation	Ligand
Progesterone receptor	PR	Progesterone
Estrogen receptor	ER	Estrogen
Liver X receptor	LXR	Oxysterols
Vitamin D3 receptor	VDR	Vitamin D3
Androgen receptor	AR	Testosterone
Glucocorticoid receptor	GR	Cortisol
Thyroid hormone receptor	TR	Thyroid hormone
Retinoic acid-related receptor	RXR	Rexinoids
Mineralocorticoid receptor	MR	Aldosterone
Peroxisome proliferator activated receptor g	PPARγ	Fatty acid Metabolites
Retinoid orphan receptor	ROR	?
Estrogen-related receptor	ERR	?

**Table 2:** Effect of different nutrients on PPARs modulation.

Nutrients	PPARs Regulation	References
**Polyunsaturated fatty acids (PUFA)**		
n-3 fatty acids	Activate both PPARα and PPARγ and lead to prevention of inflammation in adipocytes	[109,115]
n-6 fatty acid	Inhibitors of PPAR receptor signalling and regulate metabolic network	[109]
Oleoylethanolamide	Activate PPARα and induce lipolysis	[114]
Palmitoylethanolamide	Activate PPARα and provide anti-inflammatory activity	[114]
**Conjugated Linoleic acid (CLA) Isomers**		
9Z, 11E-CLA	Enhance PPAR-γ activation and exerts strong anti-cancer effects	[119,154]
10E, 12Z-CLA	Inhibits the PPAR-γ activation causing inflammation, IR and adipocyte delipidation	[155]
9Z, 11Z-CLA and 9Z, 11E-CLA	Enhanced activation of PPARβ/δ in preadipocytes	[122]
**Dietary Amino acids**		
Glutamine	Increase the expression of PPARγ and prevent metabolic stress	[125]
Arginine	Decrease the jejunal TNFa and increase the expression of PPARγ and beneficial against gut injury	[81]
**Vitamins**		
Vitamin-A [Beta Carotene (BC)]	BC supplementation can reduce the activity of PPAR-γ	[138]
Vitamin- E (Tocopherols)	α-tocopherol modulate PPAR-γ expression better than γ-tocopherol	[143,144]
1,25-dihydroxy vitamin-D3	Decrease the expression of PPAR-γ2 and regulate lipid metabolism	[152]
**Phytochemicals**		
Isoflavone	Act as a ligand for PPAR to regulate lipid metabolism	[156]
Quercetin	Inhibits the activity of all isoform of PPARs except that of PPAR-γ and prevent accumulation of fat in the liver	[119]
Lectins	Up-regulate the PPAR-γ2 and provide an adipogenic effect on mesenchymal cells	[155]
Alliin	Activates the PPAR-γ and provides a cardioprotective effect	[157]
Allicin	Inhibit the PPAR-γ2 and therefore inhibits the differentiation and inflammation of the human preadipocytes	[158]
Curcumin	Activates the PPAR-γ and confer antioxidant and anti-inflammatory activity	[159]
Resveratrol	Down-regulate PPAR-γ1−3 mRNA expression in humans and provide anti-diabetic and anti-obesity effects	[160]
Triterpenes	Suppress PPAR-γ expression and prevent cancer development	[161]
Polysaccharides	Suppress PPAR-γ expression and exert anti-cancerous activity	[161]

## Data Availability

Not applicable.
